# Crystal structure and Hirshfeld surface analysis of ketorolac tromethamine

**DOI:** 10.1107/S2056989025003226

**Published:** 2025-04-24

**Authors:** Anna M. Shaposhnyk, Vitalii V. Rudiuk, Vyacheslav N. Baumer

**Affiliations:** a Institute of Functional Materials Chemistry SSI ‘Institute for Single Crystals’ NAS of Ukraine 60 Nauky Ave Kharkiv 61001 Ukraine; b Farmak JSC, 63 Kyrylivska str., Kyiv 04080, Ukraine; Universidade de Sâo Paulo, Brazil

**Keywords:** ketorolac tromethamine, mol­ecular structure, crystal structure, Hirshfeld surface analysis

## Abstract

Ketorolac tromethamine or 1,3-dihy­droxy-2-(hy­droxy­meth­yl)propan-2-am­inium 5-benzoyl-2,3-di­hydro-1*H*-pyrrolizine-1-carboxyl­ate, C_15_H_12_NO_3_^+^·C_4_H_12_NO_3_^−^, was studied by single-crystal and powder X-ray diffraction methods. One cation and one anion are present in the asymmetric unit. In the crystal, N—H⋯O and O—H⋯O hy­dro­gen bonds link the cation and anion. All the hy­dro­gen-bond inter­actions result in the formation of a di-periodic layer in the (100) crystallographic plane.

## Abstract 2

The mol­ecular and crystal structures of ketorolac tromethamine are reported. In the crystal, mol­ecules are connected by N—H⋯O and O—H⋯O hy­dro­gen bonds, forming a two-dimensional layer in the (100) plane.

## 1. Chemical context

Ketorolac tromethamine is a non-steroidal anti-inflammatory drug (NSAID) that belongs to the class of heteroaryl acetic acid derivatives and the nonselective COX inhibitor group (Gilman, 2001 ▸).
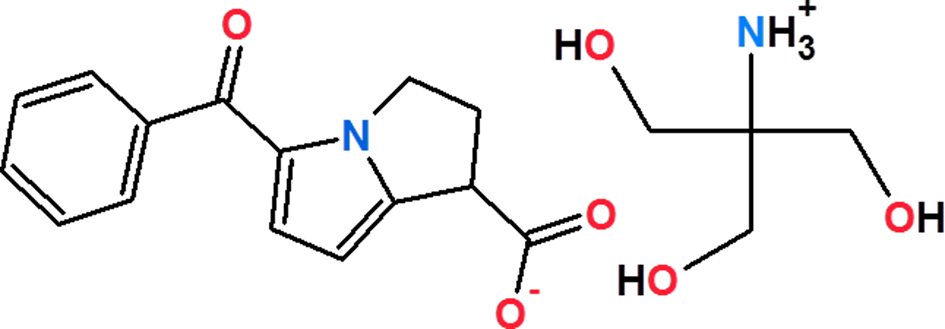


Ketorolac tromethamine produces analgesia and decreases inflammation by inhibiting the enzyme cyclo­oxygenase, resulting in a decrease in the formation of prostaglandins and sensitization to pain at sites of inflammation (Boyer *et al.*, 2010 ▸). It has also been used effectively for analgesia in advanced cancer (Joishy & Walsh, 1998 ▸). It has a chiral centre and is com­posed of (+)*R* and (−)*S* enanti­omers in equal proportions. The pharmacological (analgesic and COX inhibitory) activity is retained almost exclusively in the *S*-enanti­omer (Mroszczak *et al.*, 1990 ▸). It is commercially available as a tromethamine salt, which augments its water solubility (Litvak *et al.*, 1990 ▸) and can be given *via* routes such as intra­venous, subcutaneous, oral and intra­muscular, and is the only NSAID currently available as a nasal spray (He & Hersh, 2012 ▸). The analgesic efficacy of ketorolac depends on the racemic mixture concentrations of *S* and *R* enanti­omers (Jamali *et al.*, 1989 ▸; Mroszczak *et al.*, 1996 ▸). In the present work, we have analyzed the mol­ecular and crystal structures of ketorolac tromethamine (denoted **KT**) or 1,3-dihy­droxy-2-(hy­droxy­meth­yl)pro­pan-2-aminium 5-benzoyl-2,3-di­hydro-1*H*-pyrrolizine-1-carbox­yl­ate.

## 2. Structural commentary

**Figure 1:**
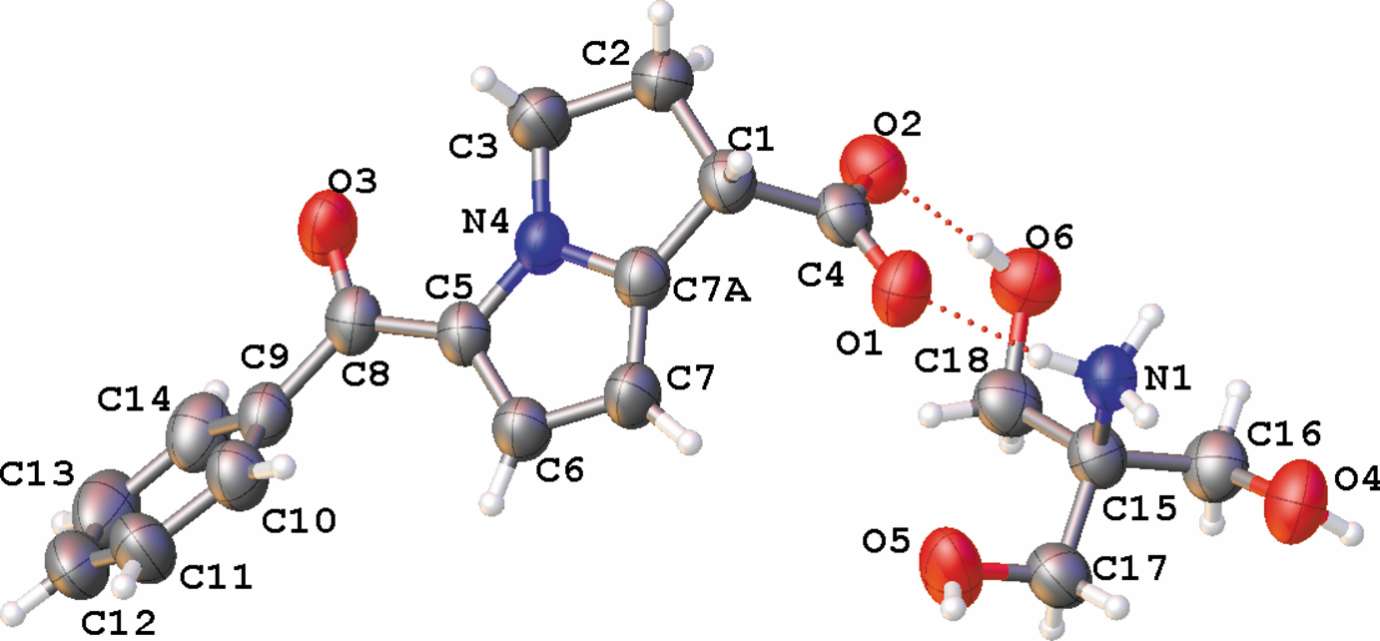
The mol­ecular structure of **KT**. Displacement ellipsoids are drawn at the 50% probability level. O—H⋯O and N—H⋯O hy­dro­gen bonds are indicated by dotted lines.

**KT** crystallizes in the monoclinic space group *I*2/*a*, with the asymmetric unit containing one anion and one cation (Fig. 1 ▸). The positive charge of the cation is located at the protonated amino group of the tromethamine mol­ecule. A result of protonation of the amino group is a lengthening of the N1—C17 distance [1.489 (3) Å] in the cation com­pared to the average C*sp*^3^—N value of 1.467 Å (Orpen *et al.*, 1994 ▸). The H atoms on atom N1 were determined from a difference Fourier map. A negative charge is located on the deprotonated carboxyl­ate group of ketorolac, as follows from the lengthening of the C4—C1 distance [1.530 (3) Å] com­pared to the average C*sp*^3^—C*sp*^2^(carb­oxy­lic acid) value of 1.502 Å (Orpen *et al.*, 1994 ▸), and the C4—O1 [1.245 (3) Å] and C4—O2 [1.258 (3) Å] distances (C—O^2−^ = 1.254 Å; Orpen *et al.*, 1994 ▸). In the 2,3-di­hydro-1*H*-pyrrolizine fragment, the saturated ring adopts an envelope conformation, where the deviation of the C2 atom from the C1/C7*A*/N4/C3 plane is 0.116 Å. The arene group of the benzaldehyde fragment is located in a +synperiplanar (+sp) position with respect to the 2,3-di­hydro-1*H*-pyrrolizine bicycle [the C6—C5—C8—C9 torsion angle is 13.0 (4)°] and is turned with respect to the C8=O3 bond [the O3—C8—C9—C14 torsion angle is 39.5 (3)°]. The carboxyl­ate group is in an equatorial position with respect to the 2,3-di­hydro-1*H*-pyrrolizine fragment and is almost coplanar with the endocyclic C1—C2 bond [the C3—C2—C1—C4 and C2—C1—C4—O2 torsion angles are 127.8 (3) and −8.1 (3)°, respectively]. The cation and anion are connected by inter­molecular N1—H1*A*⋯O1 and O6—H6*A*⋯O2 hy­dro­gen bonds (Table 1 ▸), which form a characteristic 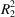
(9) graph-set motif (Etter *et al.*, 1990 ▸).

## 3. Supra­molecular features

**Figure 2:**
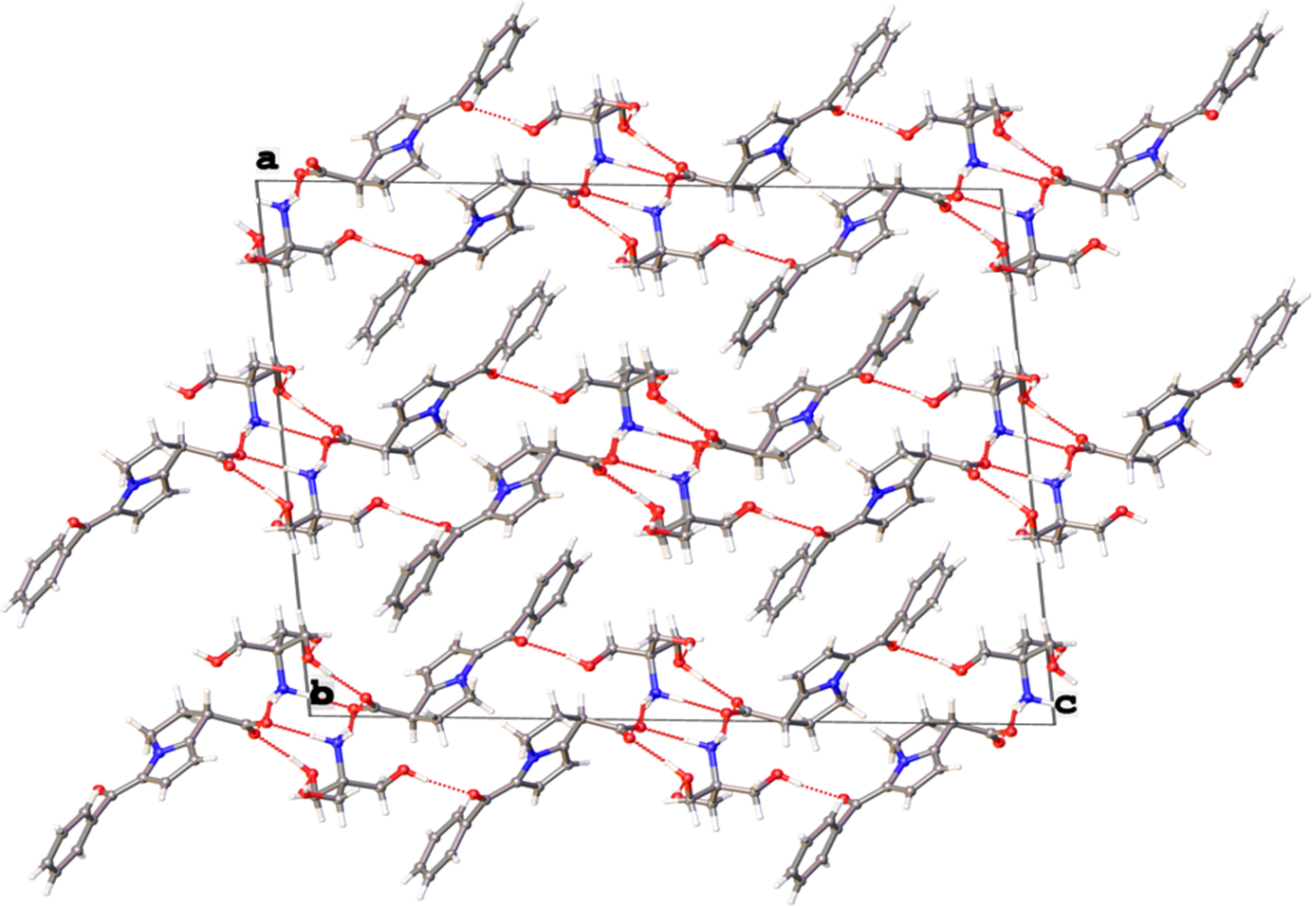
The crystal packing of **KT**, viewed along [010]. Hydrogen bonds are shown as dashed lines.

The main packing fragment in **KT** is a mono-periodic layer in the (100) plane with a characteristic 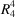
(18) graph-set motif (Etter *et al.*, 1990 ▸). In one layer, tromethamine cations form one-dimensional chains along [010], which are repeated over *a*/2 and *c*/2 *via* the O5—H5⋯O6^ii^ hy­dro­gen bonds (Table 1 ▸). One cation inter­acts with four ketorolac anions *via* a series of hy­dro­gen bonds (O4—H4⋯O3^i^, O6—H6*A*⋯O2, N1—H1*A*⋯O1, N1—H1*B*⋯O2 and N1—H1*C*⋯O1; Table 1 ▸), forming a layer in the (100) plane. Van der Waals inter­actions are observed between neighbouring layers (Fig. 2 ▸).

## 4. Database survey

A search of the Cambridge Structural Database (CSD, Version 5.44, last update June 2024; Groom *et al.*, 2016 ▸) for the 5-benzoyl-2,3-di­hydro-1*H*-pyrrolizine-1-carboxyl­ate unit resulted in one hit (CSD refcode HOJSAB; Jasinski *et al.*, 2008 ▸). In this structure, 5-benzoyl-2,3-di­hydro-1*H*-pyrrolizine-1-carboxyl­ate exists as a neutral mol­ecule. A search for the 1,3-dihy­droxy-2-(hy­droxy­meth­yl)propan-2-aminium unit or tromethamine as a cation resulted in 88 hits. 40 of these hits contain the com­pound as an anion with the carb­oxy­lic acid group deprotonated, for example, CIKQIY (Zhang *et al.*, 2013 ▸), COZBAX (Rossi *et al.*, 2020 ▸) and EDALEC (Bhattacharya *et al.*, 2012 ▸).

## 5. Synthesis and crystallization

Crystals of the title com­pound suitable for X-ray diffraction analysis were grown by recrystallization of the API ketorolac tromethamine from a water solution by the diffusion method with isopropyl alcohol at room tem­per­a­ture over a period of one week.

## 6. Hirshfeld surface analysis

Inter­molecular inter­actions can be analyzed using Hirshfeld surface analysis and 2D fingerprint plots (Turner *et al.*, 2017 ▸). Analysis and calculation of the Hirshfeld surface were carried out with *CrystalExplorer17.5* (Spackman *et al.*, 2021 ▸).

**Figure 3:**
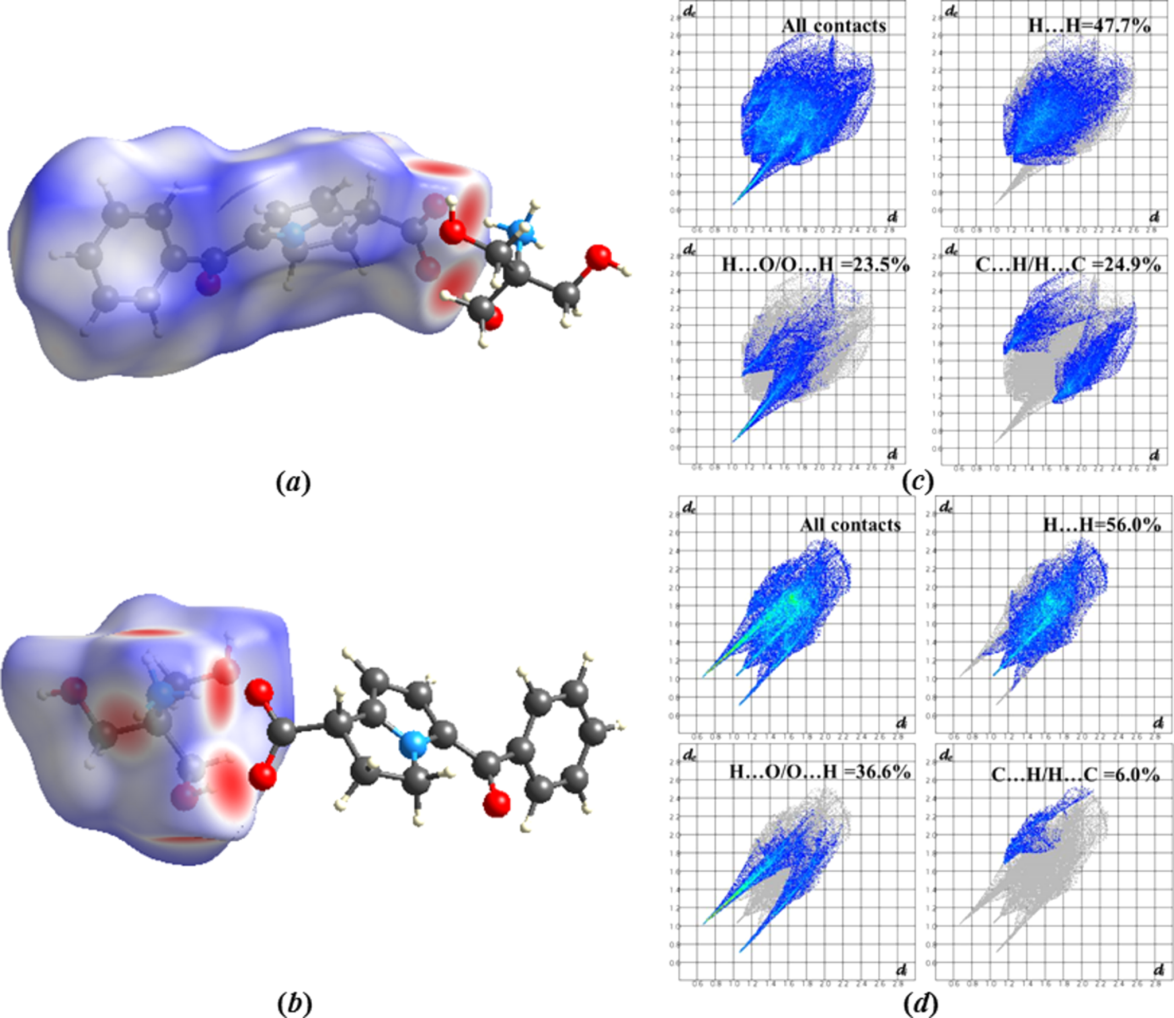
Hirshfeld surfaces mapped over *d*_norm_ for (*a*) the anion and (*b*) the cation of **KT**. Contributions of inter­actions of different types to the total Hirshfeld surface of (*c*) the anion and (*d*) the cation of **KT**.

The Hirshfeld surfaces were calculated for the structure under study using a standard high surface resolution, mapped over *d*_norm_ [Figs. 3 ▸(*a*) and 3(*b*)]. The red spots, corresponding to contacts that are shorter than the van der Waals radii sum of the closest atoms, are observed at the carboxyl­ate and carbonyl groups. To com­pare inter­molecular inter­actions of different types in a more qu­anti­tative way, their contributions to the total Hirshfeld surfaces were analysed and the main contributions are presented in Figs. 3 ▸(*c*) and 3(*d*). The main contribution for the cation and anion is provided by H⋯H short contacts. Stronger contributions of N—H⋯O and O—H⋯O hy­dro­gen bonds are observed in the structure. The contribution of C⋯H/H⋯C short contacts is significant for the anion [Fig. 3 ▸(*c*)].

## 7. Powder diffraction characterization

**Figure 4:**
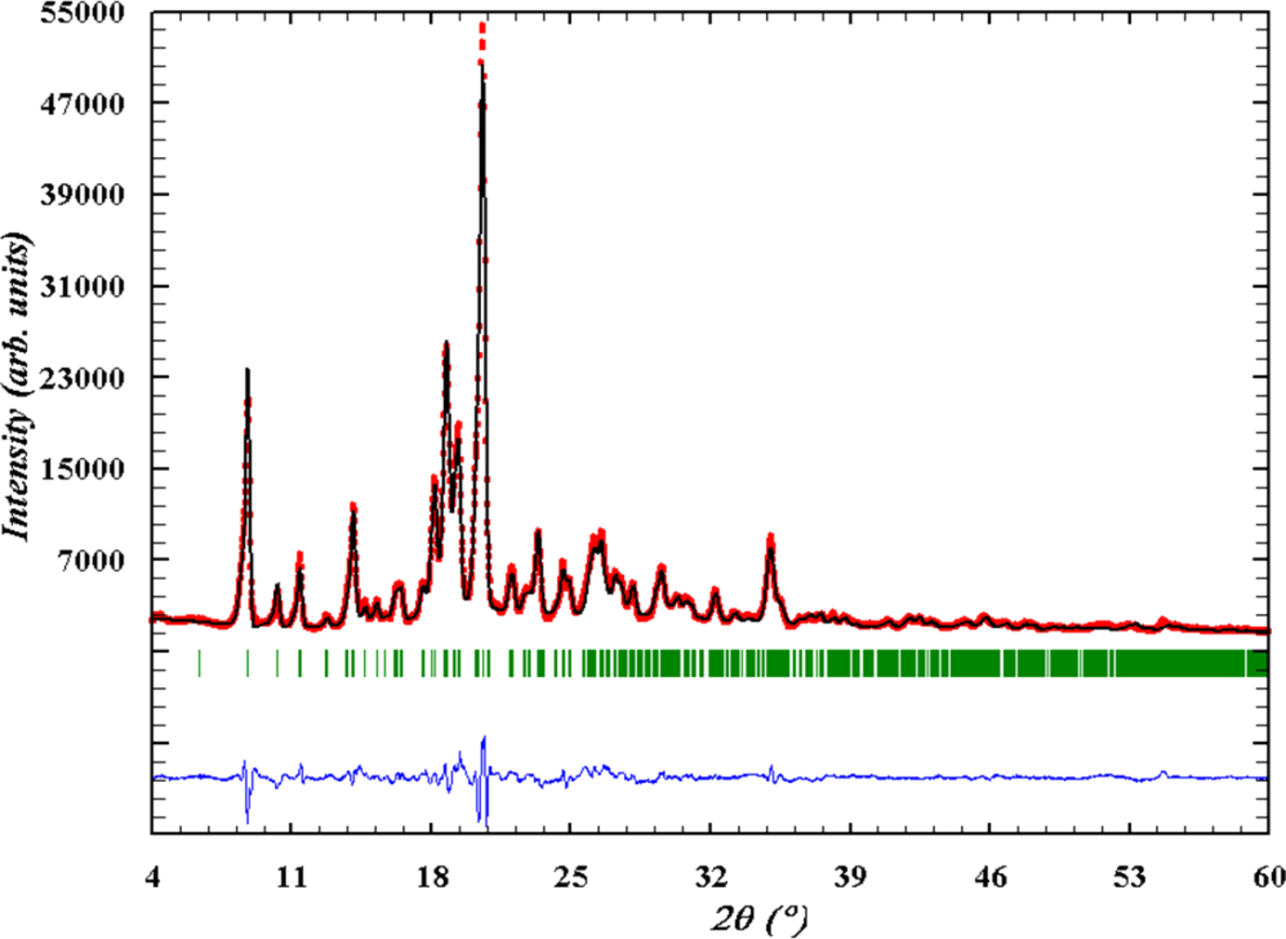
Final Rietveld plots for **KT**. Observed data points are indicated by red circles, the best-fit profile (black upper trace) and the difference pattern (blue lower trace) are shown as solid lines. The vertical green bars correspond to the Bragg reflections.

An X-ray powder diffraction pattern of **KT** was recorded using a Siemens D500 powder diffractometer (Cu *K*α radiation, Bragg–Brentano geometry, curved graphite monochromator on the counter arm, 4° < 2θ < 60°, 2θ = 0.02°). A Rietveld refinement (Fig. 4 ▸) on the basis of the obtained pattern was carried out with the *FullProf* and *WinPLOTR* programs (Rodriguez-Carvajal & Roisnel, 1998 ▸) using data of an external standard (NIST SRM1976) for the calculation of the instrumental profile function and the single-crystal data as the structure model for refinement. The main results of the Rietveld refinement are shown in Table 2 ▸. On the basis of the Rietveld refinement, the experimental powder X-ray diffraction pattern coincides with the theoretical pattern calculated from the single-crystal X-ray study.

## 8. Refinement

Crystal data, data collection and structure refinement details are summarized in Table 3 ▸. The H atoms were placed in calculated positions and treated as riding, with C—H = 0.96 Å, O—H = 0.82 Å and *U*_iso_(H) = 1.5*U*_eq_(C,O) for methyl and hydroxyl groups, and C_ar_—H = 0.93 Å (ar is aromatic), C*sp*^2^—H = 0.97 Å, N—H = 0.89 Å and *U*_iso_(H) = 1.2*U*_eq_(C,N) for all other H atoms.

## Supplementary Material

Crystal structure: contains datablock(s) I. DOI: 10.1107/S2056989025003226/ex2090sup1.cif

Structure factors: contains datablock(s) I. DOI: 10.1107/S2056989025003226/ex2090Isup2.hkl

CCDC reference: 2442550

Additional supporting information:  crystallographic information; 3D view; checkCIF report

## Figures and Tables

**Table 1:** Hydrogen-bond geometry (Å, °)

*D*—H⋯*A*	*D*—H	H⋯*A*	*D*⋯*A*	*D*—H⋯*A*
O4—H4⋯O3^i^	0.82	1.92	2.731 (3)	172
O5—H5⋯O6^ii^	0.82	1.95	2.762 (3)	174
O6—H6*A*⋯O2	0.82	1.85	2.667 (2)	177
N1—H1*A*⋯O1	0.89	1.89	2.771 (2)	172
N1—H1*B*⋯O2^iii^	0.89	2.05	2.926 (3)	169
N1—H1*C*⋯O1^iv^	0.89	1.92	2.756 (3)	155

Symmetry codes: (i) 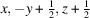; (ii) 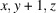; (iii) 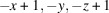; (iv) 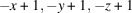.

**Table 2:** Experimental data of the X-ray powder diffraction study performed at 293 K

Crystal system, space group	Monoclinic, *I*2/*a*
*a* (Å)	20.3347 (15)
*b* (Å)	6.6301 (5)
*c* (Å)	27.981 (2)
β (°)	96.306 (4)
*V* (Å^3^)	3749.5 (5)
*D*_*x*_ (Mg m^−3^)	1.334
	
Refinement	
*R*_p_	0.0720
*R*_wp_	0.0915
*R*_exp _	0.0178
*R*_B_	0.0580
*R*_F_	0.0727

**Table 3:** Experimental details

Crystal data	
Chemical formula	C_15_H_12_NO_3_^+^·C_4_H_12_NO_3_^−^
*M*_r_	376.40
Crystal system, space group	Monoclinic, *I*2/*a*
Temperature (K)	293
*a*, *b*, *c* (Å)	20.3154 (13), 6.6466 (4), 28.0770 (15)
β (°)	96.389 (6)
*V* (Å^3^)	3767.6 (4)
*Z*	8
Radiation type	Mo *K*α
μ (mm^−1^)	0.10
Crystal size (mm)	0.56 × 0.22 × 0.07

Data collection	
Diffractometer	Rigaku Xcalibur Sapphire3
Absorption correction	Multi-scan (*CrysAlis PRO*; Rigaku OD, 2018 ▸)
*T*_min_, *T*_max_	0.584, 1.000
No. of measured, independent and observed [*I* > 2σ(*I*)] reflections	14262, 3734, 2314
*R*_int_	0.085
(sin θ/λ)_max_ (Å^−1^)	0.619

Refinement	
*R*[*F*^2^ > 2σ(*F*^2^)], *wR*(*F*^2^), *S*	0.057, 0.179, 1.04
No. of reflections	3734
No. of parameters	249
H-atom treatment	H atoms treated by a mixture of independent and constrained refinement
Δρ_max_, Δρ_min_ (e Å^−3^)	0.16, −0.19

Computer programs: *CrysAlis PRO* (Rigaku OD, 2018 ▸), *SHELXS97* (Sheldrick, 2008 ▸), *SHELXL2018* (Sheldrick, 2015 ▸) and *OLEX2* (Dolomanov *et al.*, 2009 ▸).

## supplementary crystallographic information

### 1,3-Dihydroxy-2-(hydroxymethyl)propan-2-aminium 5-benzoyl-2,3-dihydro-1H-pyrrolizine-1-carboxylate Crystal data

**Table:**

C_15_H_12_NO_3_^+^·C_4_H_12_NO_3_^−^	*F*(000) = 1600
*M**_r_* = 376.40	*D*_x_ = 1.327 Mg m^−^^3^
Monoclinic, *I*2/*a*	Mo *K*α radiation, λ = 0.71073 Å
*a* = 20.3154 (13) Å	Cell parameters from 2134 reflections
*b* = 6.6466 (4) Å	θ = 3.4–22.7°
*c* = 28.0770 (15) Å	µ = 0.10 mm^−^^1^
β = 96.389 (6)°	*T* = 293 K
*V* = 3767.6 (4) Å^3^	Prism, colourless
*Z* = 8	0.56 × 0.22 × 0.07 mm

### 1,3-Dihydroxy-2-(hydroxymethyl)propan-2-aminium 5-benzoyl-2,3-dihydro-1H-pyrrolizine-1-carboxylate Data collection

**Table:**

Rigaku Xcalibur Sapphire3 diffractometer	2314 reflections with *I* > 2σ(*I*)
Detector resolution: 16.1827 pixels mm^-1^	*R*_int_ = 0.085
ω scans	θ_max_ = 26.1°, θ_min_ = 3.2°
Absorption correction: multi-scan (CrysAlis PRO; Rigaku OD, 2018)	*h* = −23→25
*T*_min_ = 0.584, *T*_max_ = 1.000	*k* = −8→8
14262 measured reflections	*l* = −34→33
3734 independent reflections	

### 1,3-Dihydroxy-2-(hydroxymethyl)propan-2-aminium 5-benzoyl-2,3-dihydro-1H-pyrrolizine-1-carboxylate Refinement

**Table:**

Refinement on *F*^2^	0 restraints
Least-squares matrix: full	Hydrogen site location: inferred from neighbouring sites
*R*[*F*^2^ > 2σ(*F*^2^)] = 0.057	H atoms treated by a mixture of independent and constrained refinement
*wR*(*F*^2^) = 0.179	*w* = 1/[σ^2^(*F*_o_^2^) + (0.0717*P*)^2^ + 0.7854*P*] where *P* = (*F*_o_^2^ + 2*F*_c_^2^)/3
*S* = 1.04	(Δ/σ)_max_ < 0.001
3734 reflections	Δρ_max_ = 0.16 e Å^−^^3^
249 parameters	Δρ_min_ = −0.19 e Å^−^^3^

### 1,3-Dihydroxy-2-(hydroxymethyl)propan-2-aminium 5-benzoyl-2,3-dihydro-1H-pyrrolizine-1-carboxylate Special details

**Table:**

Geometry. All esds (except the esd in the dihedral angle between two l.s. planes) are estimated using the full covariance matrix. The cell esds are taken into account individually in the estimation of esds in distances, angles and torsion angles; correlations between esds in cell parameters are only used when they are defined by crystal symmetry. An approximate (isotropic) treatment of cell esds is used for estimating esds involving l.s. planes.

### 1,3-Dihydroxy-2-(hydroxymethyl)propan-2-aminium 5-benzoyl-2,3-dihydro-1H-pyrrolizine-1-carboxylate Fractional atomic coordinates and isotropic or equivalent isotropic displacement parameters (Å^2^)

**Table:**

	*x*	*y*	*z*	*U*_iso_*/*U*_eq_	
O1	0.48593 (10)	0.3303 (3)	0.44078 (6)	0.0655 (5)	
O2	0.46522 (9)	0.0085 (3)	0.42405 (6)	0.0637 (5)	
O3	0.35641 (11)	0.1436 (3)	0.20682 (6)	0.0760 (6)	
O4	0.40104 (11)	0.3531 (3)	0.61861 (7)	0.0787 (6)	
H4	0.384403	0.347567	0.643885	0.118*	
O5	0.35501 (13)	0.5730 (3)	0.48159 (7)	0.0869 (7)	
H5	0.364973	0.692435	0.483945	0.130*	
O6	0.39150 (10)	−0.0296 (3)	0.49643 (7)	0.0668 (5)	
H6A	0.415208	−0.019853	0.474738	0.100*	
N1	0.45003 (10)	0.3145 (3)	0.53307 (6)	0.0518 (5)	
H1A	0.457862	0.325858	0.502620	0.062*	
H1B	0.470895	0.206582	0.546029	0.062*	
H1C	0.464673	0.423879	0.549180	0.062*	
N4	0.42765 (10)	0.2619 (3)	0.29261 (6)	0.0528 (5)	
C1	0.50058 (13)	0.2230 (4)	0.36263 (8)	0.0555 (6)	
H1	0.542985	0.294588	0.364741	0.085 (3)*	
C2	0.50442 (16)	0.0358 (4)	0.33042 (9)	0.0688 (8)	
H2A	0.549840	0.012643	0.324234	0.085 (3)*	
H2B	0.488949	−0.082141	0.346188	0.085 (3)*	
C3	0.46117 (17)	0.0740 (4)	0.28378 (10)	0.0738 (8)	
H3A	0.487734	0.087891	0.257319	0.085 (3)*	
H3B	0.429569	−0.034252	0.276724	0.085 (3)*	
C4	0.48286 (13)	0.1827 (4)	0.41333 (9)	0.0535 (6)	
C5	0.38028 (13)	0.3803 (4)	0.26721 (8)	0.0523 (6)	
C6	0.37220 (14)	0.5445 (4)	0.29638 (9)	0.0602 (7)	
H6	0.343025	0.650623	0.288933	0.072*	
C7	0.41437 (15)	0.5256 (4)	0.33830 (9)	0.0639 (7)	
H7	0.418692	0.615070	0.363935	0.077*	
C7A	0.44886 (13)	0.3480 (4)	0.33472 (8)	0.0533 (6)	
C8	0.34637 (14)	0.3153 (4)	0.22233 (8)	0.0562 (6)	
C9	0.30014 (13)	0.4507 (4)	0.19276 (8)	0.0545 (6)	
C10	0.31445 (15)	0.6490 (4)	0.18368 (9)	0.0645 (7)	
H10	0.351325	0.709561	0.200381	0.077*	
C11	0.27477 (18)	0.7578 (5)	0.15022 (10)	0.0821 (9)	
H11	0.285832	0.889880	0.143567	0.099*	
C12	0.21897 (18)	0.6728 (6)	0.12661 (11)	0.0872 (10)	
H12	0.192488	0.746515	0.103745	0.105*	
C13	0.20222 (16)	0.4788 (7)	0.13672 (11)	0.0866 (10)	
H13	0.163240	0.423167	0.121828	0.104*	
C14	0.24326 (15)	0.3657 (5)	0.16906 (10)	0.0714 (8)	
H14	0.232702	0.232434	0.174914	0.086*	
C15	0.37749 (13)	0.2929 (4)	0.53536 (8)	0.0551 (6)	
C16	0.36743 (15)	0.2196 (4)	0.58508 (9)	0.0663 (7)	
H16A	0.320578	0.217037	0.588954	0.085 (3)*	
H16B	0.384926	0.084518	0.589999	0.085 (3)*	
C17	0.34462 (15)	0.4973 (4)	0.52700 (9)	0.0673 (7)	
H17A	0.297458	0.484927	0.529118	0.085 (3)*	
H17B	0.362784	0.590435	0.551643	0.085 (3)*	
C18	0.35096 (14)	0.1436 (4)	0.49694 (10)	0.0646 (7)	
H18A	0.306589	0.103162	0.502539	0.085 (3)*	
H18B	0.348183	0.208564	0.465850	0.085 (3)*	

### 1,3-Dihydroxy-2-(hydroxymethyl)propan-2-aminium 5-benzoyl-2,3-dihydro-1H-pyrrolizine-1-carboxylate Atomic displacement parameters (Å^2^)

**Table:**

	*U*^11^	*U*^22^	*U*^33^	*U*^12^	*U*^13^	*U*^23^
O1	0.0780 (14)	0.0680 (12)	0.0499 (10)	−0.0143 (10)	0.0042 (9)	−0.0046 (9)
O2	0.0646 (13)	0.0603 (11)	0.0655 (11)	−0.0026 (9)	0.0045 (9)	0.0066 (8)
O3	0.1105 (18)	0.0615 (12)	0.0521 (10)	0.0029 (11)	−0.0087 (10)	−0.0072 (9)
O4	0.0914 (16)	0.0912 (14)	0.0542 (11)	−0.0137 (12)	0.0111 (11)	−0.0006 (10)
O5	0.128 (2)	0.0646 (12)	0.0634 (12)	0.0050 (13)	−0.0105 (12)	0.0097 (9)
O6	0.0719 (14)	0.0568 (11)	0.0715 (12)	−0.0045 (9)	0.0081 (10)	−0.0008 (9)
N1	0.0542 (14)	0.0564 (12)	0.0435 (10)	−0.0057 (9)	−0.0004 (9)	0.0008 (8)
N4	0.0605 (14)	0.0546 (12)	0.0425 (11)	−0.0005 (10)	0.0014 (9)	−0.0023 (9)
C1	0.0518 (16)	0.0641 (15)	0.0495 (13)	−0.0002 (12)	0.0014 (11)	0.0004 (11)
C2	0.077 (2)	0.0726 (18)	0.0556 (15)	0.0166 (15)	0.0014 (14)	−0.0042 (13)
C3	0.093 (2)	0.0651 (17)	0.0601 (16)	0.0177 (16)	−0.0038 (15)	−0.0089 (13)
C4	0.0460 (15)	0.0620 (16)	0.0500 (13)	0.0002 (11)	−0.0059 (10)	0.0021 (12)
C5	0.0540 (16)	0.0590 (14)	0.0425 (12)	−0.0018 (12)	−0.0003 (11)	0.0009 (11)
C6	0.0700 (18)	0.0572 (15)	0.0514 (14)	0.0053 (13)	−0.0021 (12)	−0.0032 (11)
C7	0.078 (2)	0.0625 (16)	0.0483 (13)	0.0064 (14)	−0.0069 (13)	−0.0095 (12)
C7A	0.0583 (16)	0.0537 (14)	0.0465 (13)	−0.0026 (11)	0.0002 (11)	−0.0022 (10)
C8	0.0624 (17)	0.0608 (15)	0.0450 (13)	−0.0079 (12)	0.0043 (11)	0.0004 (11)
C9	0.0526 (16)	0.0680 (16)	0.0421 (12)	−0.0010 (12)	0.0020 (11)	−0.0045 (11)
C10	0.0650 (19)	0.0667 (17)	0.0588 (15)	−0.0027 (13)	−0.0068 (13)	−0.0028 (12)
C11	0.096 (3)	0.077 (2)	0.0690 (18)	0.0207 (18)	−0.0128 (18)	0.0017 (15)
C12	0.077 (2)	0.116 (3)	0.0642 (19)	0.033 (2)	−0.0136 (16)	−0.0076 (18)
C13	0.0513 (19)	0.139 (3)	0.0670 (19)	0.003 (2)	−0.0064 (14)	−0.022 (2)
C14	0.0592 (18)	0.095 (2)	0.0592 (16)	−0.0165 (16)	0.0048 (14)	−0.0107 (14)
C15	0.0510 (16)	0.0599 (15)	0.0525 (14)	−0.0032 (12)	−0.0029 (11)	0.0013 (11)
C16	0.0646 (19)	0.0730 (18)	0.0619 (16)	−0.0060 (14)	0.0102 (14)	0.0032 (13)
C17	0.0662 (19)	0.0673 (17)	0.0658 (17)	0.0085 (14)	−0.0042 (14)	0.0018 (13)
C18	0.0521 (17)	0.0678 (17)	0.0708 (17)	−0.0031 (13)	−0.0068 (13)	−0.0043 (13)

### 1,3-Dihydroxy-2-(hydroxymethyl)propan-2-aminium 5-benzoyl-2,3-dihydro-1H-pyrrolizine-1-carboxylate Geometric parameters (Å, º)

**Table:**

O1—C4	1.245 (3)	C5—C8	1.434 (3)
O2—C4	1.258 (3)	C6—H6	0.9300
O3—C8	1.247 (3)	C6—C7	1.382 (4)
O4—H4	0.8200	C7—H7	0.9300
O4—C16	1.413 (3)	C7—C7A	1.382 (4)
O5—H5	0.8200	C8—C9	1.486 (4)
O5—C17	1.408 (3)	C9—C10	1.379 (4)
O6—H6A	0.8200	C9—C14	1.388 (4)
O6—C18	1.416 (3)	C10—H10	0.9300
N1—H1A	0.8900	C10—C11	1.374 (4)
N1—H1B	0.8900	C11—H11	0.9300
N1—H1C	0.8900	C11—C12	1.370 (5)
N1—C15	1.489 (3)	C12—H12	0.9300
N4—C3	1.457 (3)	C12—C13	1.371 (5)
N4—C5	1.379 (3)	C13—H13	0.9300
N4—C7A	1.341 (3)	C13—C14	1.385 (4)
C1—H1	0.9800	C14—H14	0.9300
C1—C2	1.545 (4)	C15—C16	1.514 (3)
C1—C4	1.530 (3)	C15—C17	1.521 (4)
C1—C7A	1.491 (3)	C15—C18	1.520 (3)
C2—H2A	0.9700	C16—H16A	0.9700
C2—H2B	0.9700	C16—H16B	0.9700
C2—C3	1.515 (4)	C17—H17A	0.9700
C3—H3A	0.9700	C17—H17B	0.9700
C3—H3B	0.9700	C18—H18A	0.9700
C5—C6	1.385 (3)	C18—H18B	0.9700
			
C16—O4—H4	109.5	O3—C8—C5	120.1 (2)
C17—O5—H5	109.5	O3—C8—C9	118.5 (2)
C18—O6—H6A	109.5	C5—C8—C9	121.4 (2)
H1A—N1—H1B	109.5	C10—C9—C8	123.3 (2)
H1A—N1—H1C	109.5	C10—C9—C14	118.8 (3)
H1B—N1—H1C	109.5	C14—C9—C8	117.5 (3)
C15—N1—H1A	109.5	C9—C10—H10	119.7
C15—N1—H1B	109.5	C11—C10—C9	120.6 (3)
C15—N1—H1C	109.5	C11—C10—H10	119.7
C5—N4—C3	135.6 (2)	C10—C11—H11	119.9
C7A—N4—C3	113.9 (2)	C12—C11—C10	120.3 (3)
C7A—N4—C5	110.5 (2)	C12—C11—H11	119.9
C2—C1—H1	109.0	C11—C12—H12	120.0
C4—C1—H1	109.0	C11—C12—C13	119.9 (3)
C4—C1—C2	115.8 (2)	C13—C12—H12	120.0
C7A—C1—H1	109.0	C12—C13—H13	120.0
C7A—C1—C2	102.7 (2)	C12—C13—C14	120.1 (3)
C7A—C1—C4	111.2 (2)	C14—C13—H13	120.0
C1—C2—H2A	110.1	C9—C14—H14	119.9
C1—C2—H2B	110.1	C13—C14—C9	120.1 (3)
H2A—C2—H2B	108.4	C13—C14—H14	119.9
C3—C2—C1	108.0 (2)	N1—C15—C16	107.9 (2)
C3—C2—H2A	110.1	N1—C15—C17	109.1 (2)
C3—C2—H2B	110.1	N1—C15—C18	107.9 (2)
N4—C3—C2	103.5 (2)	C16—C15—C17	109.1 (2)
N4—C3—H3A	111.1	C16—C15—C18	111.8 (2)
N4—C3—H3B	111.1	C18—C15—C17	111.0 (2)
C2—C3—H3A	111.1	O4—C16—C15	107.9 (2)
C2—C3—H3B	111.1	O4—C16—H16A	110.1
H3A—C3—H3B	109.0	O4—C16—H16B	110.1
O1—C4—O2	125.0 (2)	C15—C16—H16A	110.1
O1—C4—C1	115.9 (2)	C15—C16—H16B	110.1
O2—C4—C1	119.1 (2)	H16A—C16—H16B	108.4
N4—C5—C6	105.4 (2)	O5—C17—C15	110.6 (2)
N4—C5—C8	121.5 (2)	O5—C17—H17A	109.5
C6—C5—C8	132.8 (2)	O5—C17—H17B	109.5
C5—C6—H6	125.4	C15—C17—H17A	109.5
C7—C6—C5	109.2 (2)	C15—C17—H17B	109.5
C7—C6—H6	125.4	H17A—C17—H17B	108.1
C6—C7—H7	126.7	O6—C18—C15	112.2 (2)
C7A—C7—C6	106.7 (2)	O6—C18—H18A	109.2
C7A—C7—H7	126.7	O6—C18—H18B	109.2
N4—C7A—C1	111.3 (2)	C15—C18—H18A	109.2
N4—C7A—C7	108.2 (2)	C15—C18—H18B	109.2
C7—C7A—C1	140.4 (2)	H18A—C18—H18B	107.9
			
O3—C8—C9—C10	−133.1 (3)	C6—C5—C8—O3	−168.6 (3)
O3—C8—C9—C14	39.5 (3)	C6—C5—C8—C9	13.0 (4)
N1—C15—C16—O4	53.9 (3)	C6—C7—C7A—N4	−0.8 (3)
N1—C15—C17—O5	60.4 (3)	C6—C7—C7A—C1	−179.8 (3)
N1—C15—C18—O6	45.6 (3)	C7A—N4—C3—C2	5.9 (3)
N4—C5—C6—C7	0.5 (3)	C7A—N4—C5—C6	−1.0 (3)
N4—C5—C8—O3	4.4 (4)	C7A—N4—C5—C8	−175.7 (2)
N4—C5—C8—C9	−174.0 (2)	C7A—C1—C2—C3	6.5 (3)
C1—C2—C3—N4	−7.5 (3)	C7A—C1—C4—O1	−69.9 (3)
C2—C1—C4—O1	173.4 (2)	C7A—C1—C4—O2	108.6 (3)
C2—C1—C4—O2	−8.1 (3)	C8—C5—C6—C7	174.3 (3)
C2—C1—C7A—N4	−3.0 (3)	C8—C9—C10—C11	169.7 (3)
C2—C1—C7A—C7	176.0 (3)	C8—C9—C14—C13	−172.6 (2)
C3—N4—C5—C6	−178.0 (3)	C9—C10—C11—C12	2.4 (5)
C3—N4—C5—C8	7.4 (4)	C10—C9—C14—C13	0.4 (4)
C3—N4—C7A—C1	−1.9 (3)	C10—C11—C12—C13	0.7 (5)
C3—N4—C7A—C7	178.8 (2)	C11—C12—C13—C14	−3.1 (5)
C4—C1—C2—C3	127.8 (3)	C12—C13—C14—C9	2.5 (4)
C4—C1—C7A—N4	−127.5 (2)	C14—C9—C10—C11	−2.9 (4)
C4—C1—C7A—C7	51.5 (4)	C16—C15—C17—O5	178.0 (2)
C5—N4—C3—C2	−177.2 (3)	C16—C15—C18—O6	−72.9 (3)
C5—N4—C7A—C1	−179.5 (2)	C17—C15—C16—O4	−64.5 (3)
C5—N4—C7A—C7	1.2 (3)	C17—C15—C18—O6	165.0 (2)
C5—C6—C7—C7A	0.2 (3)	C18—C15—C16—O4	172.3 (2)
C5—C8—C9—C10	45.3 (4)	C18—C15—C17—O5	−58.4 (3)
C5—C8—C9—C14	−142.0 (2)		

### 1,3-Dihydroxy-2-(hydroxymethyl)propan-2-aminium 5-benzoyl-2,3-dihydro-1H-pyrrolizine-1-carboxylate Hydrogen-bond geometry (Å, º)

**Table:**

*D*—H···*A*	*D*—H	H···*A*	*D*···*A*	*D*—H···*A*
O4—H4···O3^i^	0.82	1.92	2.731 (3)	172
O5—H5···O6^ii^	0.82	1.95	2.762 (3)	174
O6—H6*A*···O2	0.82	1.85	2.667 (2)	177
N1—H1*A*···O1	0.89	1.89	2.771 (2)	172
N1—H1*B*···O2^iii^	0.89	2.05	2.926 (3)	169
N1—H1*C*···O1^iv^	0.89	1.92	2.756 (3)	155

Symmetry codes: (i) *x*, −*y*+1/2, *z*+1/2; (ii) *x*, *y*+1, *z*; (iii) −*x*+1, −*y*, −*z*+1; (iv) −*x*+1, −*y*+1, −*z*+1.
